# Profiling and characterization of constitutive chromatin-enriched RNAs

**DOI:** 10.1016/j.isci.2022.105349

**Published:** 2022-10-13

**Authors:** Wenlong Shen, Yan Zhang, Minglei Shi, Bingyu Ye, Man Yin, Ping Li, Shu Shi, Yifei Jin, Zhang Zhang, Michael Q. Zhang, Yang Chen, Zhihu Zhao

**Affiliations:** 1 Beijing Institute of Biotechnology, No. 20, Dongdajie Street, Fengtai District, Beijing 100071, China; 2 MOE Key Laboratory of Bioinformatics, Bioinformatics Division and Center for Synthetic & Systems Biology, BNRIST/School of Medicine, Tsinghua University, Beijing 100084, China; 3 College of Life Sciences, Henan Normal University, Xinxiang 453007, China; 4 Institute of Genetics and Developmental Biology, Chinese Academy of Sciences, Beijing 100101, China; 5 The State Key Laboratory of Medical Molecular Biology, Department of Biochemistry and Molecular Biology, Institute of Basic Medical Sciences, School of Basic Medicine, Chinese Academy of Medical Sciences and Peking Union Medical College, Beijing 100005, China

**Keywords:** Biological sciences, Molecular biology, Systems biology

## Abstract

RNA species act as architectural scaffolds for nuclear structures including chromatin in eukaryotic cells. However, the composition and dynamics of tightly bound chromatin-associated RNAs during mitosis remains elusive. Here we report the identification of chromatin-enriched RNA (cheRNAs) by biochemical nuclear fractionation coupled with RNA sequencing in both interphase and mitotic phase of A549 and HeLa-S3 cell lines. We show that highly abundant cheRNAs, mostly small noncoding RNAs, are largely maintained in mitotic chromatin, and constitute a substantial part of chromatin RNA throughout cell cycle. We also show that the mitotic retained cheRNAs tend to be cell type nonspecific and might be involved in chromatin accessibility and epigenetic memory of gene expression control. Therefore, we reveal an unexpected set of cell type-nonspecific mitotic retained chromatin-enriched RNAs. We anticipate that the landscape of RNA composition of chromatin both in interphase and mitotic phase would help understanding structure and function of chromatin.

## Highlights

• Chromatin-enriched RNAs are largely maintained in mitotic cells
• Mitotic retained cheRNAs are stable abundant, enriched in small noncoding RNAs
• Mitotic retained cheRNAs tend to be cell type nonspecific
• DNA targets of mitotic retained cheRNAs in interphase remain active in mitotic phase

## Graphical abstract

**Figure:**
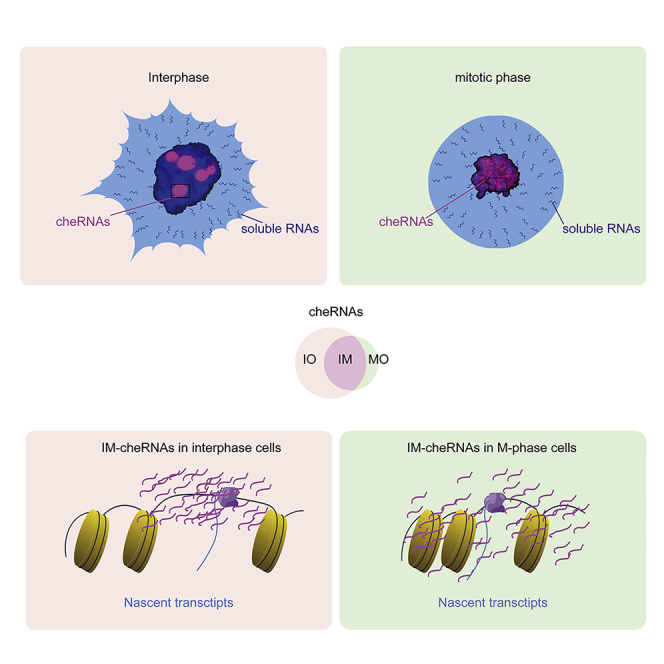

## Introduction

As important component of eukaryotic chromatin, RNAs are increasingly proposed to play diverse and essential roles in epigenetic regulation by modulating chromatin structure and states (Calandrelli et al., 2021; Hall et al., 2014; Mondal et al., 2010; Oh et al., 2021; Quinodoz et al., 2021; Thakur and Henikoff, 2020). However, the identity and function of these chromatin-associated RNAs was largely discovered in interphase cells. Whether or not these RNAs still attached to chromatin in mitotic phase remains under debate. For example, many long noncoding RNAs participating in interphase chromatin regulation detached from mitotic chromosome (Cabili et al., 2015; Sharp et al., 2020), whereas there is a perichromosomal layer composed of RNAs and proteins that contributes to chromatin remodeling during interphase (Van Hooser et al., 2005); at least some of these RNAs such as U3 small nucleolar RNAs (snoRNAs) are not dispersed during mitosis (Gautier et al., 1994), and these RNAs and proteins are proposed to various roles both in interphase and during mitosis (Hernandez-Verdun and Gautier, 1994). Therefore, it should be interesting to investigate to what extent RNAs detach from or remain bound to mitotic chromosome, and which types of RNAs might be consistently enriched in chromatin fractions.

Comprehensive profiling of cheRNAs by nuclear fractionation provided a new way to explore the identity of tightly chromatin-bound RNAs (Sun et al., 2020; Werner and Ruthenburg, 2015; Werner et al., 2017), and this strategy revealed that cell type-specific cheRNAs play import roles in gene regulation. On the other hand, profiling the mammalian mitotic chromatin-associated RNAs (mCARs) revealed noncoding RNA composition of the mitotic chromosome (Meng et al., 2016). However, the RNA types from these results are surprisingly different, probably due to differences in methods, cell lines used, and cell cycle phases. Whether mCARs are enriched or just associated with mitotic chromosome and whether cheRNAs in interphase remain specific binding to chromatin in mitotic phase remain unanswered. Therefore, the cell cycle dynamics of chromatin-enriched RNA composition are largely unknown, let alone cell type specificity of them. So, comprehensive profiling of cheRNAs both in interphase and M phase in multiple cell types is needed for better understanding of chromatin structure and regulation, especially during mitosis.

In this study, we performed biochemical fractionation of nuclei (Werner et al., 2017) coupled with RNA sequencing (RNA-seq) in both interphase and mitotic phase of A549 and HeLa-S3 cell lines. By comparing RNA abundance of chromatin and soluble fraction, we identified cheRNAs in each condition of cell cycle phases and cell lines. We show here that interphase cheRNAs are largely maintained in mitotic phase, which are largely cell type shared. These tightly chromatin-bound RNAs are highly abundant in both interphase and mitotic phase, constitute a substantial part of chromatin or chromosome RNA, and might be involved in epigenetic memory of open chromatin and active transcription in cell type-nonspecific manner. Thus, we provide landscape of RNA components of chromatin in both interphase and mitotic phase cells, and new insights into cheRNA’s housekeeping roles of regulation in chromatin accessibility and gene expression.

## Results

### Identification of cheRNAs in interphase and mitotic phase cells

**Figure 1:**
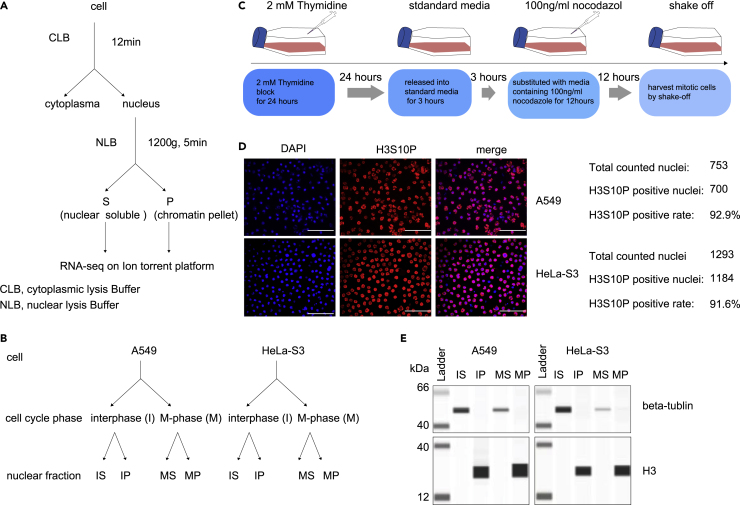
Experimental design and quality control for purification of chromatin-enriched RNAs (A) Schematic of the nuclear fractionation procedure. Purified nuclei from A549 or HeLa-S3 cells were extracted with a forcing urea/detergent buffer to yield a soluble nuclear extract (S) and chromatin pellet extract (P), and then both pools were sequenced. (B) Depiction of nuclear fractionation samples. For each cell type, we obtained two biological replicates of S and P fractions from both interphase and M-phase cells. (C) Schematic of thymidine and nocodazole treatments used to obtain early mitotic cells. (D) Immunofluorescence of anti-H3S10P- and DAPI-stained cells confirms high purity of mitotic cells following nocodazole treatment and harvest by shaking off. Scale bar, 100 μm. (E) Simple western of chromatin marker (H3) and soluble protein (beta-tubulin) confirming S and P fractions in each phase and in each cell type.

To determine binding dynamics of chromatin-enriched RNAs throughout cell cycle, we sought to apply nuclear fractionation method, which was proved to be able to separate soluble and loosely bound material from the tightly bound chromatin pellet (Bhatt et al., 2012; Werner and Ruthenburg, 2015; Werner et al., 2017), and hence to isolate cheRNAs both in interphase and M-phase of the same cell type. In this case, for A549 cells as well as HeLa-S3 cells, we performed RNA-seq for samples from two independent biological replicates of soluble nuclear extract (S) and chromatin pellet extract (P) both in asynchronized (with interphase accounting for ∼95% of cells, we called these cells as “interphase cells” afterward) and synchronized M-phase cells (Figures 1A and 1B).

Determining mitotic cheRNAs requires the pure preparations of mitotic cells. To this end, we applied method combining synchronizing and shaking to harvest pure mitotic cells (Liang et al., 2015) (Figure 1C). This method consistently yielded ∼93% mitotic cells as determined by flow cytometry and immunofluorescence-visualized anti-H3S10ph staining, or DAPI staining, to detect cells with condensed prometaphase chromatin (Figure 1D and data not shown). We confirmed released synchronized cells being able to pass through to G1 phase, ruling out the possibility that cells are undergoing apoptotic process (Figure S1). Then we confirmed our nuclear fractions by validating distribution of H3 and beta-tubulin protein in S and P fractions (Figure 1E).

To recover both short and long RNAs, we use variable read length RNA-seq technology from Ion Torrent to simultaneously sequence long and short RNAs; the read length distribution of each sample is shown in Figure S2, showing relative shorter reads are included in the libraries.

Then we applied well-described pipeline to evaluate expression of RNA genes (see STAR Methods for details, and Data S1 for statistics of sequencing data) and preformed differential expression analysis from samples aforementioned using default parameter by DESeq2 package (see STAR Methods); the intermediate results are provided as Data S2.

**Figure 2:**
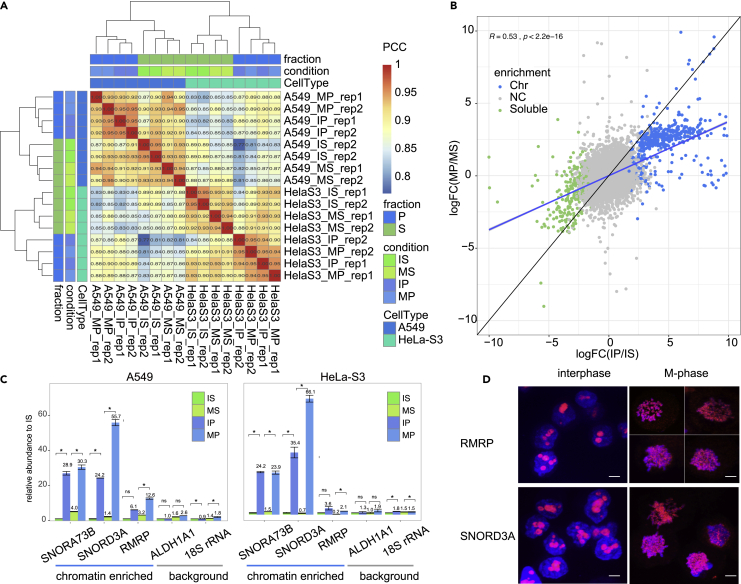
Identification of chromatin-enriched RNAs in different cell cycle phases (A) Hierarchical clustering of Pearson’s correlation based on log2-transformed RNA-seq gene counts for all the 16 samples from A549 and HeLa-S3 cells. PCC, Pearson’s correlation coefficient. BC1–16 correspond to 16 samples. (B) Scatterplot comparison between log2-transformed fold-change (logFC) of MP/MS and IP/IS in A549 cells. Color represents RNA enriched in specific nuclear fraction defined by logFC and p values calculated by DESeq2. The shaded blue line shows the linear regression curve. (C) RT-qPCR-confirmed relative abundance of selected IM_CERs and control in specific samples. 18s rRNA served as control. The numbers indicated abundances normalized to IS for each RNA. Data are represented as mean ± SEM. Comparisons were made between indicated groups with two-sided Wilcoxon rank-sum test. ∗p < 0.05. (D) RNA fluorescence *in situ* hybridization shows localization of RMRP and SNORD3A in interphase and mitotic phase Hela-S3 cells. Scale bar, 5 μm.

The first question is whether RNA composition of chromatin is drastically changed during mitosis. To this end, Pearson’s correlation based on log-transformed read counts is calculated and clustered in Figure 2A. Strikingly, the samples from same fraction (P or S fraction) clustered closer than from same cell phase, indicating similar RNA composition of chromatin in interphase and M-phase in either A549 or HeLa-S3 cells.

Consistently, log-transformed fold change of P fraction versus S fraction in interphase (logFC(IP/IS)) is correlated with that in M-phase (logFC(MP/MS)) (Pearson’s r = 0.53 in A549 cells and 0.38 in HeLa-S3 cells, p < 2.2 × 10^−16^) (Figures 2B and S3A), indicating that cheRNAs in interphase tend to be also enriched on chromatin in M-phase.

We identified cheRNAs using default parameters (logFC > 1 and false discovery rate < 0.05) in interphase and mitotic phase cells, respectively. This led to identification of overlapped interphase (I-phase) and mitotic phase (M-phase) cheRNAs (Figure S3B). These RNAs tightly bind to chromatin both in interphase and M-phase, which we termed “IM-cheRNAs.” In the meantime, RNAs that are defined as cheRNAs only in interphase are termed “interphase only” (IO-cheRNAs), whereas those defined only in M-phase are termed “M-phase only” (MO-cheRNAs). The whole list of these cheRNAs is provided in Data S3. Although we obtained IM-, IO-, and MO- cheRNAs as mentioned earlier, heatmap based on log2-transformed read counts confirmed enrichment of cheRNAs on chromatin, which does not appear to be cell cycle specific (Figure S3C). The aforementioned results suggested that these cheRNAs are similarly enriched in chromatin fraction both in interphase and mitotic phase.

Among the cheRNA lists are some well-known chromatin-associated RNAs, such as RMRP and SNORD3A (Data S2) (Quinodoz et al., 2021). We then confirmed enrichment on chromatin pellet (P) fraction of three cheRNAs by RT-qPCR (Figure 2C); RMRP and SNORD3A localization on chromatin was also confirmed by RNA fluorescence *in situ* hybridization (Figure 2D).

### Tightly bound cheRNAs constitute constant part of chromatin both in interphase and mitotic phase

**Figure 3:**
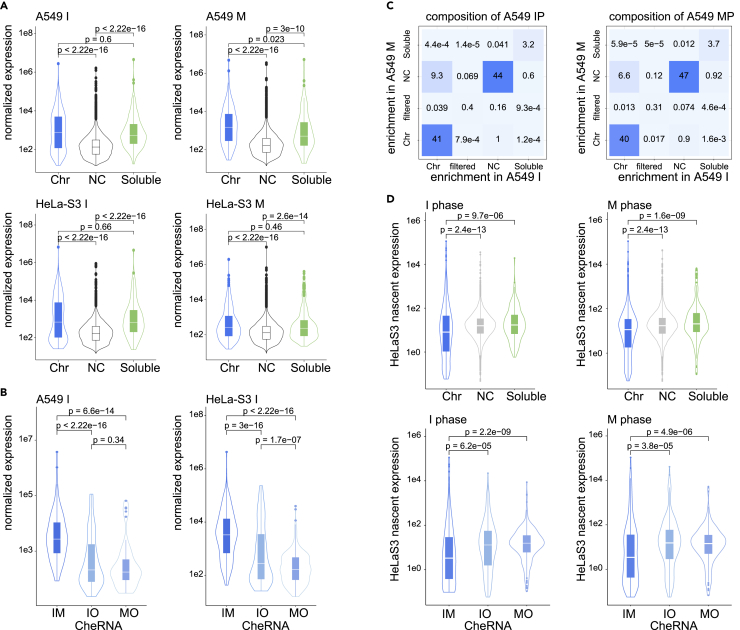
cheRNAs are highly abundant and stably associated with chromatin (A) Normalized expression (baseMean called by DESeq2) of chromatin enriched (Chr), Soluble enriched (Soluble), or negative background (NC) RNAs in interphase and M-phase of A549 and HeLa-S3 cells. Boxplots show largest (upper whisker), smallest (lower whisker), 50% quantile (center), upper hinge (75% quantile), and lower hinge (25% quantile). Comparisons were made between indicated groups with two-sided Wilcoxon’s rank-sum test. (B) Normalized expression of different groups of cheRNAs. IM: Chromatin-enriched RNAs in both interphase and M-phase, IO: RNAs only enriched in interphase chromatins, MO: RNAs only enriched in M-phase chromatins. Boxplots show largest (upper whisker), smallest (lower whisker), 50% quantile (center), upper hinge (75% quantile), and lower hinge (25% quantile). Comparisons were made between indicated groups with two-sided Wilcoxon’s rank-sum test. (C) Composition of chromatin-associated RNAs (CARs) in A549 interphase and M-phase cells. Each RNA is categorized based on its enrichment in particular cell cycle phase. “Filtered” means RNAs with low abundance that were filtered out before DESeq2 comparisons. Numbers and colors illustrated percentage of given category of RNAs. (D) Normalized nascent expression (rpm) of different groups of enriched RNAs in HeLa-S3 interphase and M-phase cells. Boxplots show largest (upper whisker), smallest (lower whisker), 50% quantile (center), upper hinge (75% quantile), and lower hinge (25% quantile). Comparisons were made between indicated groups with two-sided Wilcoxon’s rank-sum test.

We compared abundance of these cheRNAs with background RNAs, and found interestingly that both chromatin-enriched and soluble-enriched RNAs are more abundant than background (Figure 3A). Specifically, mitotic retained cheRNAs (IM-cheRNAs) are even more abundant (Figure 3B). This leads us to ask how much do cheRNAs account for total RNA component of chromatin. We counted total reads of interphase and M-phase chromatin fractions grouped by nuclear fraction enrichment, and found strikingly that about 40% of chromatin-associated RNAs are chromatin enriched both in interphase and M-phase (Figure 3C). These results indicate that tightly bound cheRNAs constitute an important and constant part of chromatin both in interphase and mitotic phase.

Recent studies suggested roles of nascent transcription in chromatin structure remodeling (Creamer et al., 2021; Finn and Misteli, 2019; Li and Fu, 2019; Quinodoz et al., 2021). To check if these cheRNAs are by-products of nascent transcription, we re-used nascent RNA-seq data performed in HeLa-S3 cells (Liang et al., 2015). As results, we found nascent transcription of cheRNAs are not higher but lower than background or soluble enriched RNAs, and mitotic retained ones (IM-cheRNAs) are even lower. This result suggests that cheRNAs, especially IM-cheRNAs, are not temporarily attached to chromosomes due to nascent transcription, but rather functional RNAs that tightly bound to chromatin throughout cell cycle.

Next, we asked if these cheRNAs are enriched in any unique classes of RNA. We compared exon numbers and transcript length of these RNAs, and found cheRNAs are significantly shorter, with less exons (Figures S4A and S4B). We used HGNC dataset to annotate these RNA genes, and found cheRNAs are enriched in snoRNAs and small nuclear RNAs (snRNAs) (Figure S4C), and the proportion of these small RNAs are even higher in IM-cheRNAs (Figure S4D). In contrast, soluble RNAs are enriched in tRNAs, which are cytoplasmic RNAs (Figure S4C), further confirming specificity of nucleic fractionation methods. It was reported that snoRNAs have relative long half-lives (Tani et al., 2012), consistent to their stable association with chromatin. Recent study suggested pre-mRNA splicing is a crucial process that allows proper progression of the cell cycle (Petasny et al., 2021); here snRNAs as cheRNAs might contribute to this process.

Previous studies suggested that RNAs with repetitive elements such as long interspersed nuclear elements (LINEs) and short interspersed nuclear elements (SINEs) associated with euchromatic interphase chromosomes (Hall et al., 2014; Percharde et al., 2018; Lu et al., 2021). We intersected exons of cheRNAs with RepeatMasker annotation track on the UCSC human genome browser and found both IM-cheRNAs and IO- or MO-cheRNAs contain repetitive RNAs, whereas at the same time, IM-cheRNAs contains less SINEs, LINEs, than IO-cheRNAs (Figure S4E), suggesting that IM-cheRNAs are generally distinct with LINE and SINE RNAs.

### Chromatin enriched RNAs activate DNA targets

Considering these stable and tight cheRNAs occupy a substantial component of chromatin-associated RNA, our next question is their potential function. Here, we use previous RNA-DNA interaction data, MARGI (Sridhar et al., 2017) and GRID (Li et al., 2017), to infer the chromatin-binding sites of these RNAs.

**Figure 4:**
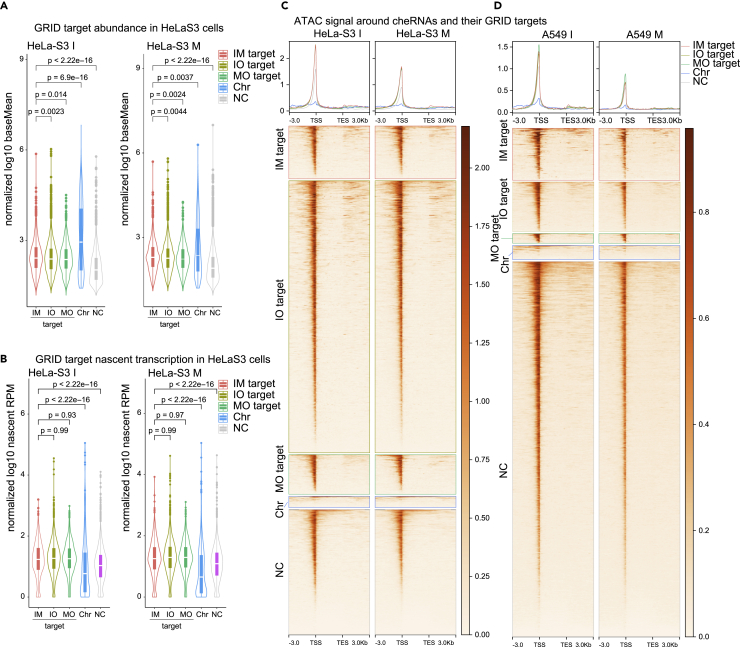
cheRNAs are associated with open chromatin and active transcription (A) Comparisons of cheRNAs and their GRID targets abundance in HeLa-S3 cells. IM, IO, and MO indicate GRID targets (see STAR Methods) of respective cheRNAs, whereas “Chr” indicates cheRNAs themselves. Boxplots show largest (upper whisker), smallest (lower whisker), 50% quantile (center), upper hinge (75% quantile), and lower hinge (25% quantile). Comparisons were made between indicated groups with two-sided Wilcoxon’s rank-sum test. (B) Comparisons of nascent expression (log10 RPM) cheRNAs and their GRID targets in HeLa-S3 cells. RNAs are indicated as in (A). Boxplots show largest (upper whisker), smallest (lower whisker), 50% quantile (center), upper hinge (75% quantile), and lower hinge (25% quantile). Comparisons were made between indicated groups with two-sided Wilcoxon’s rank-sum test. (C) ATAC signal around cheRNAs and their GRID targets in HeLa-S3 cells. RNAs are same indicated as in (A). (D) ATAC signal around cheRNAs and their GRID targets in A549 cells. IM, IO, and MO indicate GRID targets (see STAR Methods) of respective cheRNAs in A549 cells, whereas “Chr” indicates cheRNAs themselves.

First, we compared abundance of RNAs in our nuclear fractionation data and found that both cheRNAs themselves and their GRID target genes are more abundant than background in HeLa-S3 cells (Figure 4A). Similar results are also observed in A549 cells and HeLa-S3 cells when comparing cheRNAs and their MARGI target genes to background (Figure S5). In other words, we observed higher abundance of target genes of cheRNAs inferred by either GRID or MARGI methods in A549 cells and HeLa-S3 cells.

Then, we compared nascent transcription of both cheRNAs and their target genes. Intriguingly, although nascent transcription of cheRNAs is lower than background (Figures 3D and 4B), all of their GRID or MARGI target gene transcriptions are higher in both A549 and HeLa-S3 cells (Figures 4B and S6). These data suggest that these cheRNAs might be associated with higher expression of their target genes.

Furthermore, to investigate whether cheRNAs are involved in open chromatin we performed ATAC-seq assay in both interphase and M-phase of A549 and HeLa-S3 cells. Consistent to nascent transcription results, we observed less ATAC signals around cheRNA transcription start sites (TSSs) than background control, whereas even higher signals around TSS of their GRID (Figures 4C and 4D) and MARGI target genes (Figure S7). Interestingly, we did not observe significant difference in both nascent transcription and ATAC signal among targets of IM-, IO-, and MO-cheRNAs. Altogether, although cheRNAs are less transcribed, their binding to chromatin might associate with open chromatin and higher nascent transcription, both in interphase and M-phase cells.

### Chromatin-enriched RNAs are shared among cell types

Finally, we asked if cheRNAs are cell type specific or shared. We intersected cheRNAs from individual cell cycle phase and cell line, and found large fractions of cheRNAs are shared between samples (Figure S3D), although there are some cell type- and cell cycle phase-specific cheRNAs under standard cutoff. The largest intersection set of cheRNAs are actually shared in all the four conditions (g1 group in Figure S3D). More importantly, cheRNAs shared by more conditions is generally more abundant and more tightly bound to chromatin than specific ones (Figure S8). To make conclusion clearer, we selected cheRNAs that are shared in both cell cycle phases in A549, whereas in neither phase in HeLa-S3 as “A549 specific,” and vice versa, that are shared in both cell cycle phases in HeLa-S3, whereas in neither phase in A549 as “HeLa-S3 specific” (g7 and g8 groups in Figure S8).

**Figure 5:**
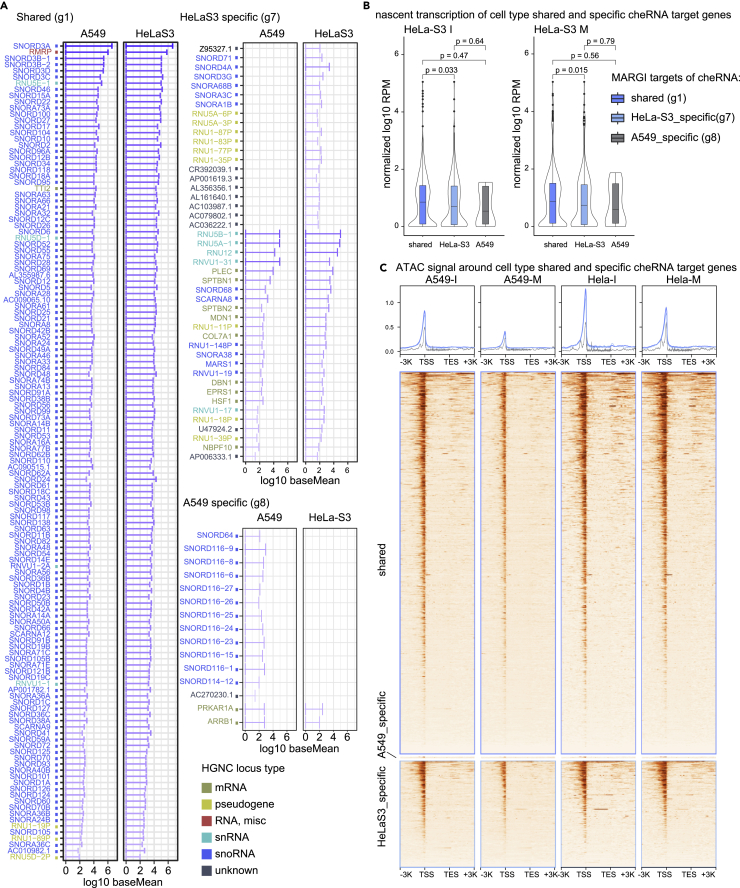
Cell type-shared cheRNAs are more prominent (A) baseMean of individual cell type-shared and -specific cheRNAs selected from Figure S3D. Color indicates HGNC locus type annotation of these RNAs. (B) Nascent transcription of cell type-shared and -specific cheRNA target genes. Boxplots show largest (upper whisker), smallest (lower whisker), 50% quantile (center), upper hinge (75% quantile), and lower hinge (25% quantile). Comparisons were made between indicated groups with two sided Wilcoxon’s rank-sum test. (C) ATAC signal around cell type-shared and -specific cheRNA target genes.

As expected, cell type-shared cheRNAs, largely snoRNAs, are much more abundant (Figures S8 and 5A) than cell type-specific ones. There are some cell type-specific cheRNAs that are only expressed in one cell type (Figure 5A). Nascent transcription of target genes of shared cheRNAs is higher than that of HeLa-S3-specific cheRNAs target genes, whereas we did not observe significant differences between shared and A549-specific cheRNAs target genes’ nascent transcription, possibly because of the limited number of A549-specific cheRNAs (Figure 5B). Consistently, ATAC signal are also slightly higher around cell type-shared cheRNAs target genes TSS than cell type-specific cheRNAs target genes. These data all suggest that cell type-shared cheRNAs play more prominent roles than cell type-specific ones.

## Discussion

### Chromatin-enriched RNAs as constant part of RNA composition of chromatin

Owing to lack of high-throughput data for comparing cheRNAs at different stages in the same cell, it was once a mystery whether RNA was retained on the M-phase chromosome. In this study, we performed nuclear fractionation RNA-seq in both interphase and mitotic phase of A549 and HeLa-S3 cells, and found a group of highly abundant and tightly bound chromatin-enriched RNAs. These RNAs constitute a constant part of chromatin, which are tightly bound to chromatin throughout cell cycle. Interestingly, since chromatin enrichment represented by logFC are correlated between interphase and mitotic phase (Figures 2B and S3A), although there are some IO- and MO-cheRNAs under standard thresholds, heatmap of read counts from all the samples did not tell significant differences between these cheRNAs (Figure S3C). Target of these cheRNAs are also similarly active (Figure 4). It is possible that IO- and MO-cheRNA tend to be also chromatin enriched throughout cell cycle, to less extent. The constant and abundant cheRNAs might help samples from chromatin pellet cluster together, irrespective of cell cycle (Figure 2A).

Accumulating evidences have suggested that although chromosomes condense 2- to 3-fold during mitosis, chromatin accessibility and binding of transcription factors (TFs) to mitotic chromatin is partially preserved (Caravaca et al., 2013; Dey et al., 2009; Ginno et al., 2018; Hsiung et al., 2015; Kadauke et al., 2012; Teves et al., 2016). And primary polymer structure of chromatin was relatively stable in mitotic phase (Ou et al., 2017). Recent analysis suggested that histone marker could also serve as either tissue-specific or nonspecific bookmarking factors (Liu et al., 2017). Here we provide data suggesting that cheRNAs are also largely retained, and hence support the notion that mitotic retention is the rule rather than the exception and may thus contribute to the inheritance of epigenetic information (Ginno et al., 2018).

### Mitotic retained cheRNAs are enriched in small noncoding RNAs

Through the analysis of gene length, number of exons, and HGNC classification, it is observed here that tightly bound cheRNAs are enriched in small noncoding RNAs such as snoRNAs and snRNAs (Figure S4). And proportion of snoRNA is even higher for cell type-shared ones. This is consistent to significant enrichment of snoRNAs in mouse mCARs (Meng et al., 2016).

It is quite interesting that previous nuclear fractionation study on interphase cells did not mention these small noncoding RNAs. A possible explanation is due to size selection of library construction and downstream analysis in previous reports, which removed transcripts shorter than 200 or 1000 bases for analysis (Sun et al., 2020; Werner and Ruthenburg, 2015; Werner et al., 2017), while many of cheRNAs from this study are shorter than 200 bases (Figure S4B).

It is worth noting that albeit with relative low nascent transcription, the cheRNAs are highly abundant, occupying a considerable part of the total chromatin RNA. It suggests that these RNAs are very stable and might play roles both in interphase and mitotic phase.

Recent studies have proposed more roles of snoRNAs apart from canonical roles in rRNA processing. Here in this study, we show that cheRNAs, especially cell type-shared ones, are highly enriched in snoRNAs, expanding possible roles of them in modulating chromatin accessibility and gene expression control, along with other possible roles, such as rebuilding nucleolus in G1 (discuss below).

### Cell type specificity of cheRNAs

In this study, we propose that cheRNAs are mainly composed of cell type-nonspecific RNA, although we do not rule out the existence of cell type-specific ones because we were able to find both cell cycle- and cell type-specific cheRNAs under standard thresholds. However, the heatmap of these RNAs in our nuclear fractionation dataset (Figure S3C) does not discriminate different types of cheRNAs. On the other hand, cell type-shared cheRNAs are more abundant and more tightly chromatin bound (Figure S8), and cell type-shared cheRNAs seem to play more prominent roles in chromatin accessibility and transcription control (Figure 5).

We noted that previous works revealed cell type-specific chromatin-enriched long noncoding RNAs mark active and repressive *cis*-regulation, which are different from the cheRNAs in this study. Thanks to recovering small RNAs in the nuclear fractionation method, cheRNAs in this study are enriched in small noncoding RNAs. Although we identified some cheRNAs in interphase that are specific to cell lines (group g13 and g14 in Figure S3D), chromatin enrichment of these kinds of cheRNAs seem to reduce in M-phase (Figure S8B). These RNAs are with relatively low abundance and might not contribute much to RNA composition of chromatin (Figure S8A). Therefore, it is likely that mitotic retained cheRNAs are a distinct group of chromatin-associated RNAs, which might play different roles from previously reported ones. It would be interesting to investigate different roles and mechanisms of different type of cheRNAs.

### Possible roles of cheRNAs in chromatin accessibility and transcriptional control

Recently, increasing evidence suggests RNAs as facilitators of functional genomic interactions (Li and Fu, 2019; Oh et al., 2021). Although nuclear fractionation RNA-seq does not provide direct evidence for roles of these cheRNAs, we inferred the DNA target genes of these RNAs reusing RNA-DNA interaction data obtained in multiple cells and by multiple methods. We compared abundance, nascent transcription, and chromatin accessibility of TSS regions of these target genes with background controls and obtained all consistent results: the chromatin targets of cheRNAs are accessible and nascent transcriptions are more active and hence abundance is also higher than background controls. Therefore, it is reasonable to speculate that these RNAs are associated with open chromatin and active transcription. At this stage, it is difficult to judge whether the functions of these RNAs in M phase and interphase are the same. The RNA localization pattern in interphase and M-phase chromatin seems apparently different, which diffusely covers the chromosomes in the M phase, whereas shows a patchy distribution in interphase (Figure 2D). Therefore, the mitotic binding site of IM-cheRNAs are probably different with their interphase targets, although due to lack of data and methods, precise DNA targets of these cheRNAs in mitotic cells are unavailable at this stage. However, interphase chromatin targets of cheRNAs are more open (Figures 4C and 4D) and nascent transcription is still prominent (Figure 4B) than negative controls in mitotic cells, suggesting roles of these cheRNAs in epigenetic memory on gene expression control. It was proposed that RNA should be considered an as integral component of nuclear organization and these protein/RNA structures form a dynamic nuclear mesh that can regulate interphase chromatin structure (Nozawa and Gilbert, 2019). At the same time, a specialized chromosome domain called the perichromosomal layer was formed in mitotic cells (Booth et al., 2014; Sun and Kaufman, 2018; Van Hooser et al., 2005); this layer comprises 30%–47% of the entire chromosome volume (Booth et al., 2016) and contains proteins and RNAs that are important for proper function, such as cell cycle progression. In this study, we identified SNORD3A, also known as U3 snoRNA, is highly enriched in chromatin both in interphase and mitotic chromosomes. This RNA was long proposed as a perichromosomal layer component (Gautier et al., 1994). There are many similar snoRNAs in the IM-cheRNA list, especially cell type-nonspecific ones. It is reasonable that these RNAs might also play roles in establishing and maintaining proper chromatin structure, possibly through phase separation, which should be carefully elucidated in the future. It will be interesting to further investigate whether there is any functional relevance between roles in gene expression and rebuilding chromatin and nucleolar structure as perichromosomal layer. Further efforts are still needed to integrate.

### Limitations of the study

Owing to lack of high-throughput RNA-DNA interaction data exact target sites of IM-cheRNA are not available currently. This study found that interphase target gene of these IM-cheRNAs remained relatively active in M phase, which led to speculation that these RNAs were related to the epigenetic memory that controls gene expression. However, on the other hand, these RNAs seem to exhibit different chromatin-binding patterns in M- and I-phases, and previous studies have found that at least some of them overlap with perichromosomal layer RNAs, which are critical for rebuilding nucleolar structure in G1 phase. Therefore, more effective systems biology methods are needed to clarify the interrelationships between functions of RNAs in the remodeling of nucleolar structure and the remodeling of gene expression.

## STAR★Methods

### Key resources table

**Table:** 

REAGENT or RESOURCE	SOURCE	IDENTIFIER
**Antibodies**		

beta Tubulin	abcam	ab6046
Histone H3	abcam	ab1791
Goat Anti-Rabbit IgG HRP	cwbiotech	CW0103S
Goat Anti-Mouse IgG HRP	cwbiotech	CW0102S
Histone H3 S10P	abcam	ab5176

**Deposited data**		

The ATAC-seq and nucleic fractionation RNA-seq data	this paper	GEO: GSE195694
GRID data	GEO	GEO: GSE82312
MARGI data	GEO	GEO: GSE92345
HeLa-S3 nascent transcription sequence data	GEO	SRA: SRP062167
Scripts for idenfifying cheRNAs and other analysis	This study	https://doi.org/10.5281/zenodo.7024571

**Experimental models: Cell lines**		

A549	ATCC	CCL-185
HeLa-S3	ATCC	CCL-2.2

**Software and algorithms**		

R v3.5.3	R Foundation	https://cran.r-project.org
Python v2.7	Python Software Foundation	https://www.python.org
*HISAT2*	https://github.com/DaehwanKimLab/hisat2	http://daehwankimlab.github.io/hisat2/
*Samtools*	http://www.htslib.org	https://github.com/samtools/
StringTie	The Center for Computational Biology at Johns Hopkins University	https://ccb.jhu.edu/software/stringtie/
DESeq2	https://bioconductor.org/packages/DESeq2/	https://bioconductor.org/packages/DESeq2/

### Resource availability

#### Lead contact

Further information and requests for resources and reagents should be directed to and will be fulfilled by the lead contact, Zhihu Zhao (zhaozh@bmi.ac.cn).

#### Materials availability

This study did not generate new unique reagents.

### Experimental model and subject details

#### Cell lines and cell culture

A549 (ATCC CCL-185) and HeLa-S3 (ATCC CCL-2.2) Cells were grown in standard DME/F12 1:1 media (HyClone #SH30023.01) with 10% Bovine serum (Gibco #16170078), 1% PenStrep (Gibco #15140). 48 hours prior to synchronization, cells were plated on 15 cm plates at 50% confluence. First, cells were incubated with media containing 2 mM Thymidine (Sigma T1895-10G) for 24 hours, washed twice with 10 mL 1x PBS and released into standard media for 3 hours. Media were then substituted with media containing 100 ng/mL nocodazole (Sigma #M1404) for 12 h. At the end of the incubation, media with floating mitotic cells was collected.

### Method details

#### Nuclear fractionation RNA-seq

Nuclear fractionation of A549 and HeLa-S3 cells were performed following (Bhatt et al., 2012), with minor modifications: 10 mM Ribonucleoside Vanadyl Complex (NEB) was added to all the buffers to prevent RNA degradation.

Total RNA from each sample were rRNA depleted with Ribominus kit (Thermofisher), and total RNA-seq libraries were constructed with Ion Total RNA-Seq libray kit V2 (Thermofisher). Libraries were then sequenced on an Ion Proton sequencer (Thermofisher).

#### ATAC-seq

ATAC-seq was performed following a previously published protocol (Buenrostro et al., 2015). The libraries were sequenced on illumina sequencers to obtain 2 × 150 paired-end reads.

ATAC-seq data were aligned to hg38 using Bowtie2 with parameters “--local --very-sensitive-local --no-unal --no-mixed --no-discordant -I 10 -X 700”. In addition, PCR duplicates were discarded. Biological replicates were combined for further presentation. DeepTools were used to produce bigWig tracks for ATAC-seq signals, and create heatmaps for scores associated with genomic regions.

#### Simple Western

The nucleic fractionation samples and reagent were subsequently loaded into an assay plate and placed in a ProteinSimple JES system (JS3106; ProteinSimple, San Francisco, CA, USA.) according to the standard protocol. The resulting chemiluminescent signal was detected and quantitated by ProteinSimple Compass software, using antibodies listed in the key resources table.

#### Stellaris RNA FISH

Stellaris® custom Stellaris® FISH Probes were designed against RMRP(NR_003051) and SNORD3A(NR_006880) by utilizing the Stellaris® RNA FISH Probe Designer (Biosearch Technologies, Inc., Petaluma, CA) available online at www.biosearchtech.com/ stellarisdesigner (version 4.2). The cells were hybridized with the Stellaris RNA FISH Probe set labeled with CAL Fluor® Red 590 Dye (Biosearch Technologies, Inc.), following the manufacturer’s instructions available online at www.biosearchtech.com/stellarisprotocols. Briefly, the cell is adhered onto a #1 coverglass and permeabilized with 70% ethanol. Hybridization was completed overnight at +37°C in a generic laboratory incubator. After hybridization, wash buffers with short incubation periods are used to remove excess probes. The sample was imaged using confocal microscopy.

#### Immunofluorescence staining

For mitotic cell purity analyses, the mitotic cells were plated onto cleaned-up coverslips and fixed in 4% paraformaldehyde for 10 minutes. Then the coverslips were washed with PBS and permeabilized for 10 min with 0.5% Triton X-100 in PBS. The cells were incubated with Anti-Histone H3 (phospho S10) antibody (ab5176) (diluted 1:100) and after blocking with 3% bovine serum albumin for 1 h. The cells were then incubated with Goat Anti-Rabbit IgG H&L (Alexa Fluor® 594 ab150077, for Anti-Histone H3 (phospho S10) and Alexa Fluor® 488 ab150140, for gamma H2A.X phospho S139) (diluted 1:200). The DNA is stained with DAPI (blue). The immunofluorescence was analyzed under a Perkin-Elmer Ultraview Confocal Imaging System.

For DNA damage analysis, cells were pretreated as above and were incubated with Anti-gamma H2A.X (phospho S139) antibody (ab2893) (diluted 1:100) after blocking with 3% bovine serum albumin for 1 h. The cells were then incubated with Goat Anti-Rabbit IgG H&L (Alexa Fluor® 488) (ab150077) (diluted 1:200). The DNA is stained with DAPI (blue). The immunofluorescence was analyzed under a Perkin-Elmer Ultraview Confocal Imaging System.

#### Defining cheRNAs

Raw reads from Ion proton sequencer were evaluated gene expression followed by (Pertea et al., 2016). Briefly, reads were mapped to hg38 (GRCh38.p13) using hisat2 (Kim et al., 2015), and used stringtie (Pertea et al., 2015) with gencode.v33.chr_patch_hapl_scaff.annotation.gtf (https://www.gencodegenes.org/human/release_33.html) to evaluate abundance of transcripts and genes. We also tried to assemble transcripts using stringtie, and evulate abundance of gene expression based on stringtie assembled transcript, we found vast majority of reads are annotated, and cheRNAs are largely annotated genes. We decided to use genecode annotation instead of assembled transcripts.

Gene count data for each sample were subjected to DESeq2 package in R (Love et al., 2014). We identified cheRNAs in each individual phase one by one cell line. We only took genes with read per million (rpm)>1 in at least 2 samples into subsequent analysis, this resulted in ∼13,000 genes passed through the filter in each condition. Log(FC)>1, and FDR<0.05 was used as threshold to classifying cheRNAs, then interphase cheRNAs overlapped with M-phase cheRNAs were defined as IM-cheRNAs.

Exon count and gene length information is based on Genecode v33 gtf as above. Gene type and family were annotated based on The HUGO Gene Nomenclature Committee (HGNC) complete dataset download from HGNC website (ftp://ftp.ebi.ac.uk/pub/databases/genenames/new/tsv/hgnc_complete_set.txt).

Detailed scripts and parameters could be found at github, and DOIs are listed in key resources table.

#### Public data HeLa-S3 nascent transcription integrative analysis

HeLa-S3 nascent transcription sequence data were download from GEO (https://www.ncbi.nlm.nih.gov/sra?term=SRP062167) by SRA toolkit. Gene abundance evaluation were performed as above. Then nascent transcription of different group of RNAs were compared by Mann-Whitney’ test. Correlation between nascent transcription and chromatin enrichment were tested by pearson correlation tests.

#### Public GRID and MARGI data integrative analysis

Processed GRID data was downloaded from GEO (GSE82312), Processed MARGI data was downloaded from GEO (GSE92345), BED tools intersect was used for inferring DNA targets of cheRNAs. Genes ovelapped with cheRNAs target DNA regions were considered as target genes.

All the scripts used in data analysis were deposited to github, DOI was listed in key resources table.

### Quantification and statistical analysis

#### Statistical analysis

Fisher’s extact test was performed to test enrichment of HGNC gene type in each group of cheRNAs. For quantitative comparison between different groups, Mann-Whitney-Wilcoxon test was performed. p < 0.05 was considered statistically significant, and in all figures, ∗ represents p < 0.05. Multiple test was corrected by Benjamini and Hochberg method. Correlation tests were performed in R using pearson’s method.

## Data Availability

The ATAC-seq and nucleic fractionation RNA-seq data generated in this study have been deposited at GEO (GSE195694) and are publicly available as of the date of publication. Accession numbers are listed in the key resources table. All original code has been deposited at Zenodo and is publicly available as of the date of publication (https://doi.org/10.5281/zenodo.7024571). DOIs are listed in the key resources table. Any additional information required to reanalyze the data reported in this paper is available from the lead contact upon request.

## Supplemental information

Document S1. Figures S1–S8

Data S1. Mapping statics of all the samples, related to Figure 2

Data S2. DESeq2 statistics for enrichment of genes in nuclear fraction, related to Figures 2 and 3 Column description: baseMean: mean of normalized counts for all samples; log2FoldChange: log2 fold change; lfcSE: SE stat: Wald statistic; pvalue: Wald test p values; padj: Benjamini & Hochberg adjusted p values, diection: enrichment on chromatin or soluble extracts

Data S3. The whole list of cheRNAs in BED format, related to Figures 3 and 5 Column description: columns 1–12 correspond to standard BED12 format, column13: information from genecode gtf, column 14: gene symbol, column 15: transcript length; column 16: type of cheRNAs: one of “IO,” “MO,” or “IM” cheRNAs, column 17: HGNC locus type, column 18: HGNC locus group.
