# Supplementary material for: Profiling and characterization of constitutive chromatin-enriched RNAs
Source: iScience. 2022 Oct 13;25(11):105349. doi: 10.1016/j.isci.2022.105349 (PMC9618790; doi:10.1016/j.isci.2022.105349)
Supplement: Document S1. Figures S1–S8 [file mmc1.pdf]

iScience, Volume *25*

Supplemental information

Profiling and characterization of constitutive chromatin-enriched RNAs

Wenlong Shen, Yan Zhang, Minglei Shi, Bingyu Ye, Man Yin, Ping Li, Shu Shi, Yifei Jin, Zhang Zhang, Michael Q. Zhang, Yang Chen, and Zhihu Zhao

Supplementary Figure Legends

Figure S1. Morphology and flow cytometry of A549 cells released from synchronizing. Related to Figure 1.

(A) Morphology images taken from series of time after releasing into fresh media.

(B) Cell cycle phase as assessed by flow cytometry.

(C) Stacked barplot showing proportion of each cell cycle phase at indicated hours after releasing to fresh media.

Figure S2. Read length distribution of RNA-seq reads. Related to Figure 2.

Histogram of read length from each individual sample, showing median of reads.

Figure S3. Identification of CheRNAs in A549 and HeLa-S3 cells. Related to Figure 2.

(A) Scatter plot comparison between log2 transformed fold-change (logFC) of MP/MS and IP/IS in HeLa-S3 cells. Color represented RNA enriched in specific nuclear fraction defined by logFC and P values calculated by DESeq2. The shaded blue line shows the linear regression curve.

(B) Venn plots show overlaps of cheRNAs from interphase and M-phase in A549 and HeLa-S3 cells.

(C) Heatmap shows Log transformed readcount of each cheRNA in each sample.

(D) Upset plots shows intersections of cheRNAs from each sample. All the 15 groups of cheRNAs are then categorized based on number of shared samples. For clarity, cell type shared cheRNAs (g1) and cell type specific cheRNAs (g7 for HeLa-S3 specific and g8 for A549 specific) are selected as indicated.

Figure S4. cheRNAs are enriched in small ncRNAs. Related to Figure 3.

(A) Empirical cumulative distribution of exon counts of nuclear fraction enriched RNAs or background RNAs.

(B) Density plot of transcript length of nuclear fraction enriched RNAs or background RNAs.

(C) Heatmap of statistic (odds ratio) of hypergeometric test different nuclear enriched RNAs are enriched in different HGNC types of RNAs.

(D) Proportion of HGNC types of RNA in each group of cheRNAs.

(E) Proportion of repetitive RNAs in different nuclear fraction enriched of RNAs.

(F) Proportion of repetitive RNAs in different type of cheRNAs.

Figure S5. cheRNAs targets are more abundant. Related to Figure 4.

(A) Comparisons of HeLa-S3 cheRNAs and their targets (inferred from HEK MARGI data) abundance in HeLa-S3 cells. IM, IO and MO indicate MARGI targets (see methods) of respective cheRNAs. While “Chr”indicates cheRNAs themselves. Comparisons were made between indicated groups with two sided wilcox rank sum test.

(B) Same as (A) except that cheRNA targets are inferred from H9 MARGI data.

(C) Comparisons of A549 cheRNAs and their targets (inferred from HEK MARGI data) abundance in A549 cells. IM, IO and MO indicate MARGI targets (see methods) of respective cheRNAs. While “Chr”indicates cheRNAs themselves. Comparisons were made between indicated groups with two sided wilcox rank sum test.

(D) Same as (C) except that cheRNA targets are inferred from H9 MARGI data.

Figure S6. cheRNAs targets are more transcribed. Related to Figure 4.

Nascent RNA-seq of cheRNAs and their targets in HeLa-S3 cells

(A) Comparisons of nascent transcription (log10 RPM) of cheRNAs and their targets (inferred from HEK MARGI data) in HeLa-S3 cells. IM, IO and MO indicates MARGI targets of respective cheRNAs. While “Chr”indicates cheRNAs themselves. Comparisons were made between indicated groups with two sided wilcox rank sum test.

(B) Same as (A), except that target genes are inferred from H9 MARGI data.

Figure S7. ATAC signal around cheRNA and targets. Related to Figure 4.

ATAC signal around cheRNAs and their MARGI targets in A549 and HeLa-S3 cells. IM, IO and MO indicate MARGI targets of respective cheRNAs as indicated. While “Chr”indicates cheRNAs themselves.

Figure S8. Cell type shared cheRNAs are more abundant and chromatin enriched. Related to Figure 5.

(A) Overall abundance (baseMean) of different groups of cheRNAs, as annotated in figure S2 in each indicated sample.

(B) Chromatin enrichment (logFC) of different groups of cheRNAs, as annotated in figure S2 in each indicated sample.

Figure S1

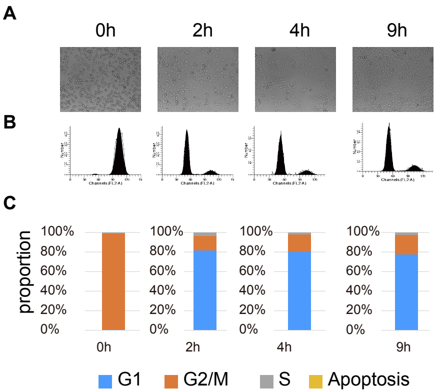
A
0h
2h
4h
9h
B
C
proportion
100%
80%
60%
40%
20%
0%
0h
2h
4h
9h
G1
G2/M
S
Apoptosis

Figure S2

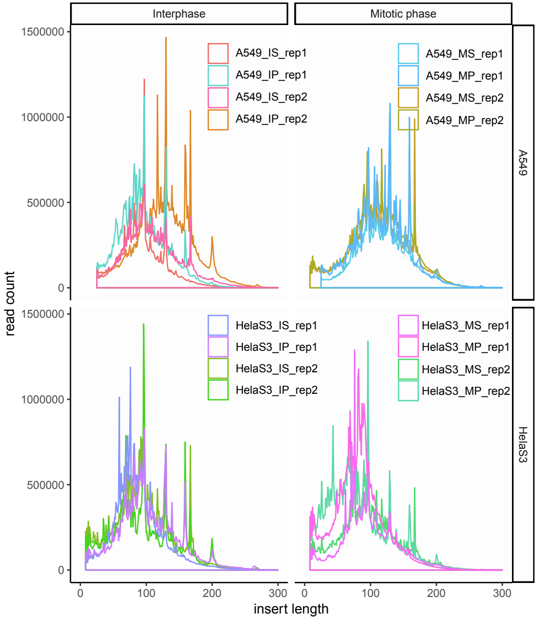

Interphase
Mitotic phase
A549
HelaS3
read count
insert length
1500000
1000000
500000
0
0
100
200
300
A549_IS_rep1
A549_IP_rep1
A549_IS_rep2
A549_IP_rep2
A549_MS_rep1
A549_MP_rep1
A549_MS_rep2
A549_MP_rep2
HelaS3_IS_rep1
HelaS3_IP_rep1
HelaS3_IS_rep2
HelaS3_IP_rep2
HelaS3_MS_rep1
HelaS3_MP_rep1
HelaS3_MS_rep2
HelaS3_MP_rep2

Figure S3

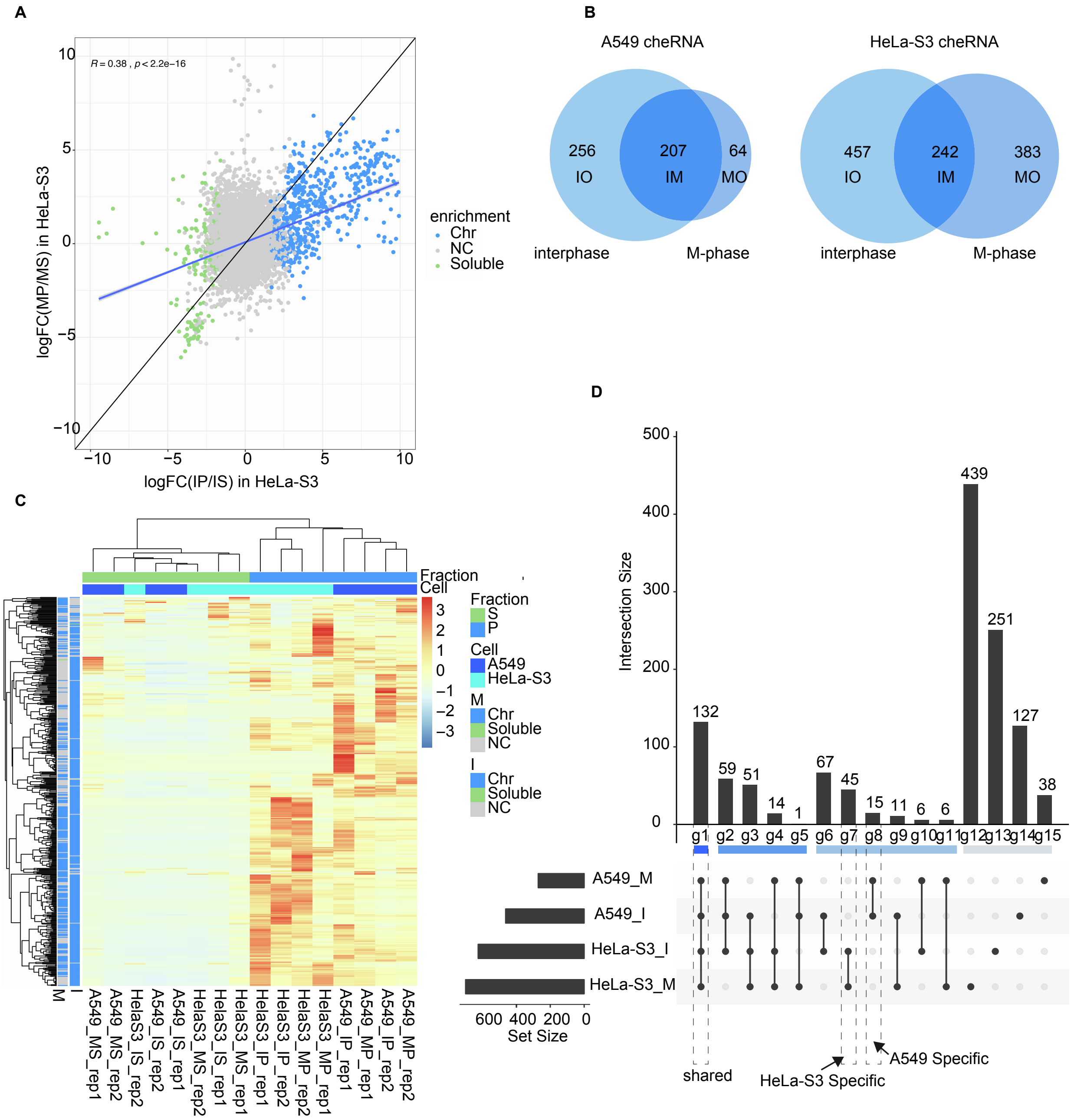
A
R = 0.38 , p < 2.2e−16
logFC(MP/MS) in HeLa-S3
logFC(IP/IS) in HeLa-S3
enrichment
Chr
NC
Soluble
B
A549 cheRNA
256
IO
207
IM
64
MO
interphase
M-phase
HeLa-S3 cheRNA
457
IO
242
IM
383
MO
interphase
M-phase
C
Fraction
Cell
Fraction
S
P
Cell
A549
HeLa-S3
M
Chr
Soluble
NC
I
Chr
Soluble
NC
A549_MS_rep1
A549_MS_rep2
HelaS3_IS_rep2
A549_IS_rep2
A549_IS_rep1
HelaS3_MS_rep2
HelaS3_IS_rep1
HelaS3_MS_rep1
HelaS3_IP_rep1
HelaS3_IP_rep2
HelaS3_MP_rep2
HelaS3_MP_rep1
A549_IP_rep1
A549_MP_rep1
A549_IP_rep2
A549_MP_rep2
D
Intersection Size
132
59
51
14
1
67
45
15
11
6
6
439
251
127
38
g1 g2 g3 g4 g5 g6 g7 g8 g9 g10 g11 g12 g13 g14 g15
A549_M
A549_I
HeLa-S3_I
HeLa-S3_M
Set Size
shared
HeLa-S3 Specific
A549 Specific

Figure S4

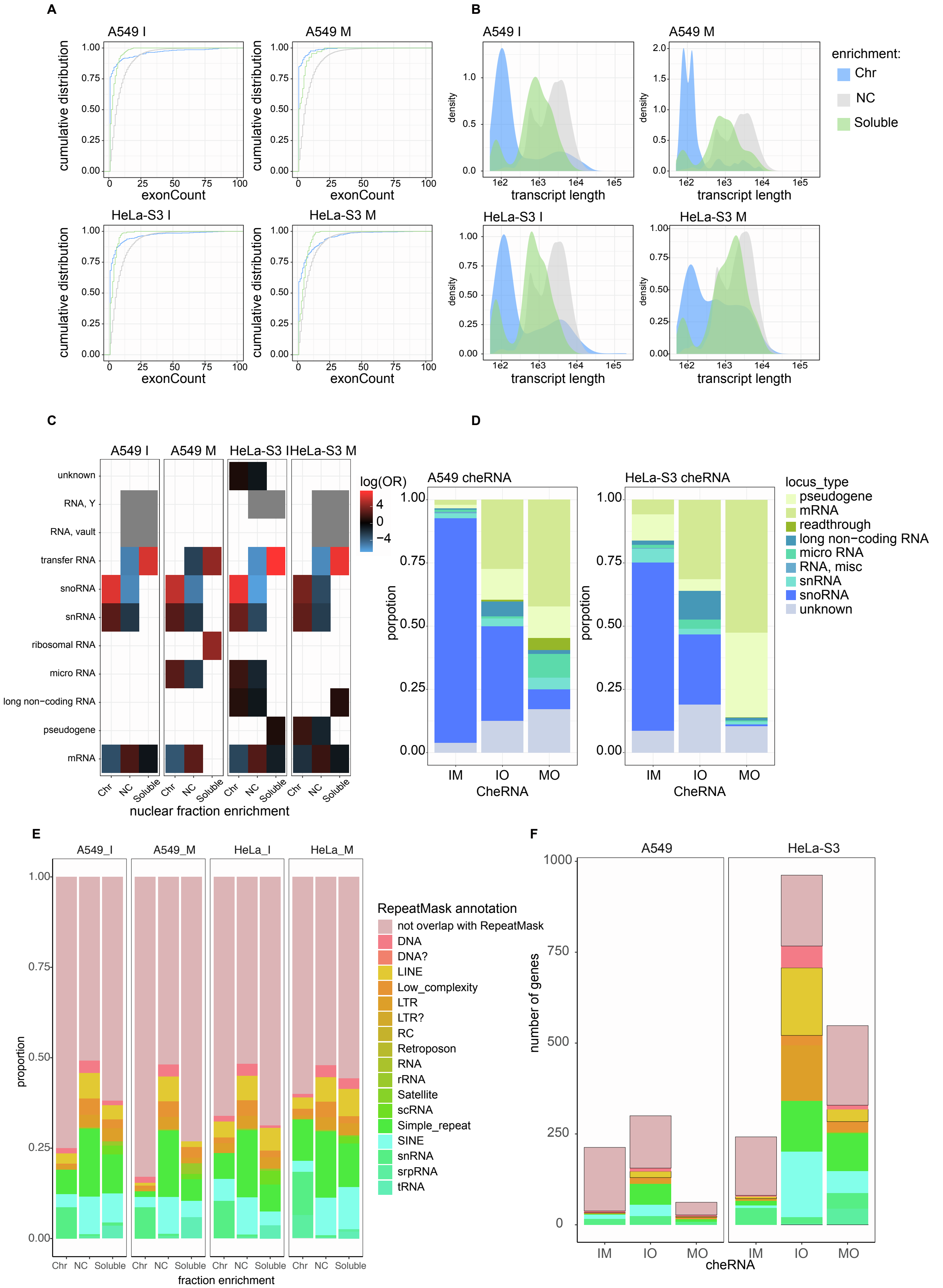

A
A549 I
A549 M
HeLa-S3 I
HeLa-S3 M
cumulative distribution
exonCount
B
enrichment:
Chr
NC
Soluble
density
transcript length
C
unknown
RNA, Y
RNA, vault
transfer RNA
snoRNA
snRNA
ribosomal RNA
micro RNA
long non−coding RNA
pseudogene
mRNA
log(OR)
Chr
NC
Soluble
nuclear fraction enrichment
D
A549 cheRNA
HeLa-S3 cheRNA
porpotion
IM
IO
MO
CheRNA
locus_type
pseudogene
mRNA
readthrough
long non−coding RNA
micro RNA
RNA, misc
snRNA
snoRNA
unknown
E
A549_I
A549_M
HeLa_I
HeLa_M
proportion
RepeatMask annotation
not overlap with RepeatMask
DNA
DNA?
LINE
Low_complexity
LTR
LTR?
RC
Retroposon
RNA
rRNA
Satellite
scRNA
Simple_repeat
SINE
snRNA
srpRNA
tRNA
fraction enrichment
F
A549
HeLa-S3
number of genes
cheRNA

Figure S5

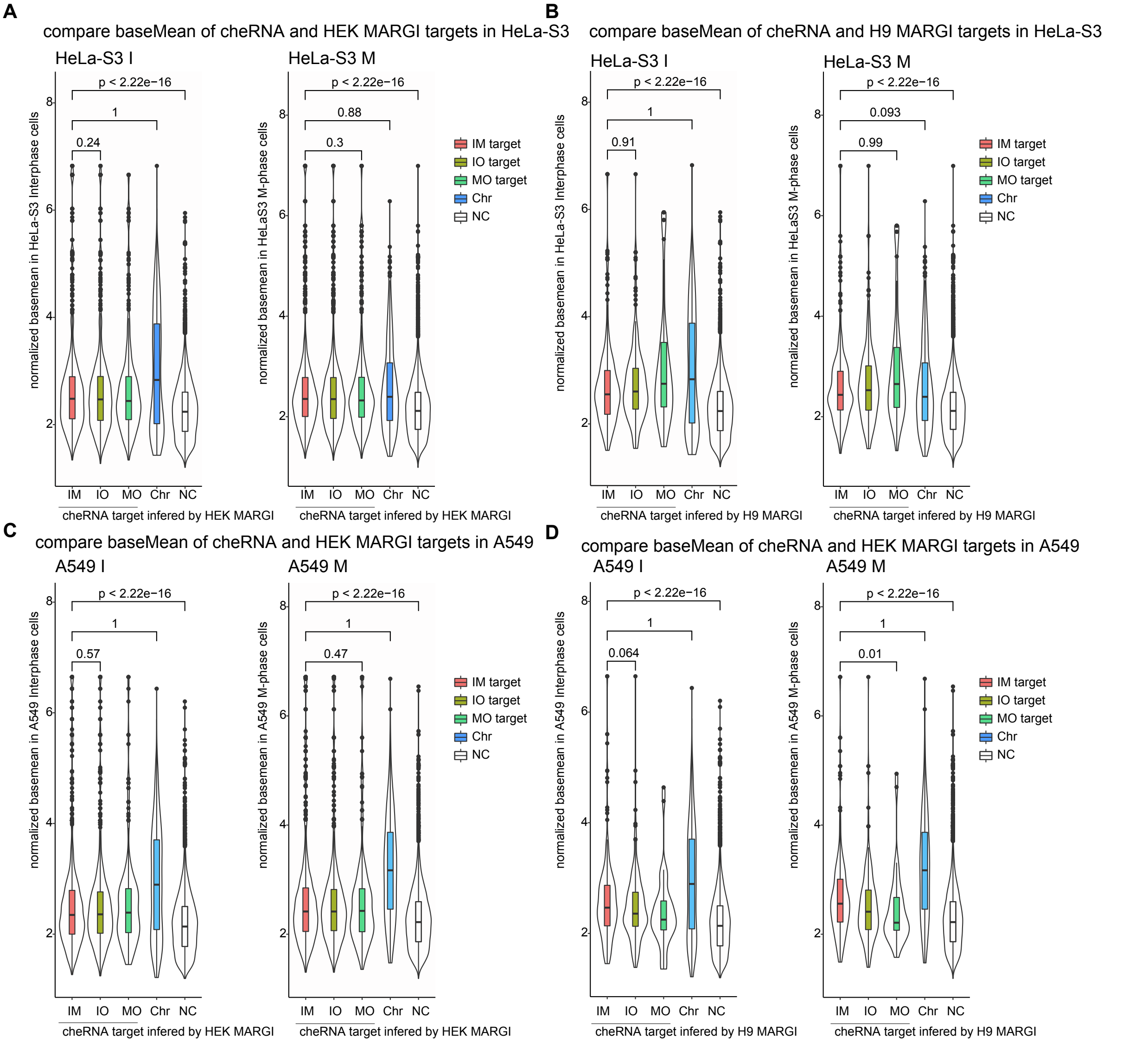
A
compare baseMean of cheRNA and HEK MARGI targets in HeLa-S3
HeLa-S3 I
HeLa-S3 M
normalized basemean in HeLa-S3 Interphase cells
normalized basemean in HeLaS3 M-phase cells
p < 2.22e−16
1
0.24
0.88
0.3
IM target
IO target
MO target
Chr
NC
IM IO MO Chr NC
cheRNA target infered by HEK MARGI
B
compare baseMean of cheRNA and H9 MARGI targets in HeLa-S3
0.91
0.093
0.99
cheRNA target infered by H9 MARGI
C
compare baseMean of cheRNA and HEK MARGI targets in A549
A549 I
A549 M
normalized basemean in A549 Interphase cells
normalized basemean in A549 M-phase cells
0.57
0.47
D
compare baseMean of cheRNA and HEK MARGI targets in A549
0.064
0.01

Figure S6

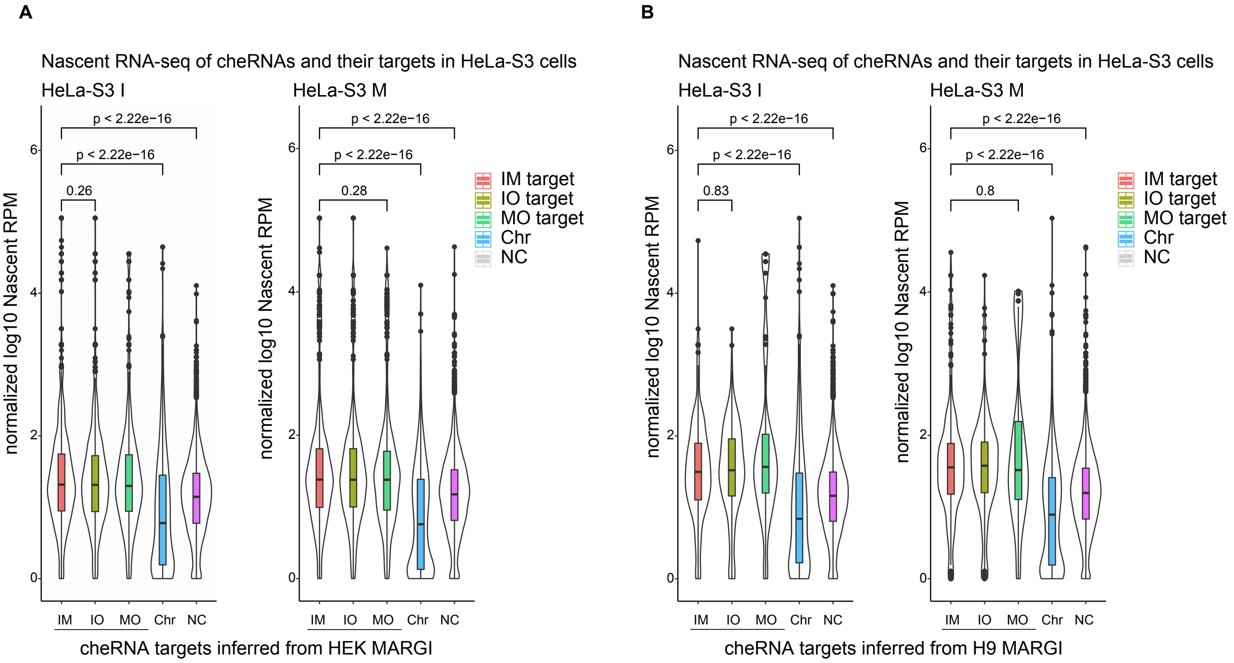
A
B
Nascent RNA-seq of cheRNAs and their targets in HeLa-S3 cells
HeLa-S3 I
HeLa-S3 M
p < 2.22e−16
0.26
0.28
0.83
0.8
normalized log10 Nascent RPM
IM target
IO target
MO target
Chr
NC
IM
IO
MO
cheRNA targets inferred from HEK MARGI
cheRNA targets inferred from H9 MARGI

Figure S7

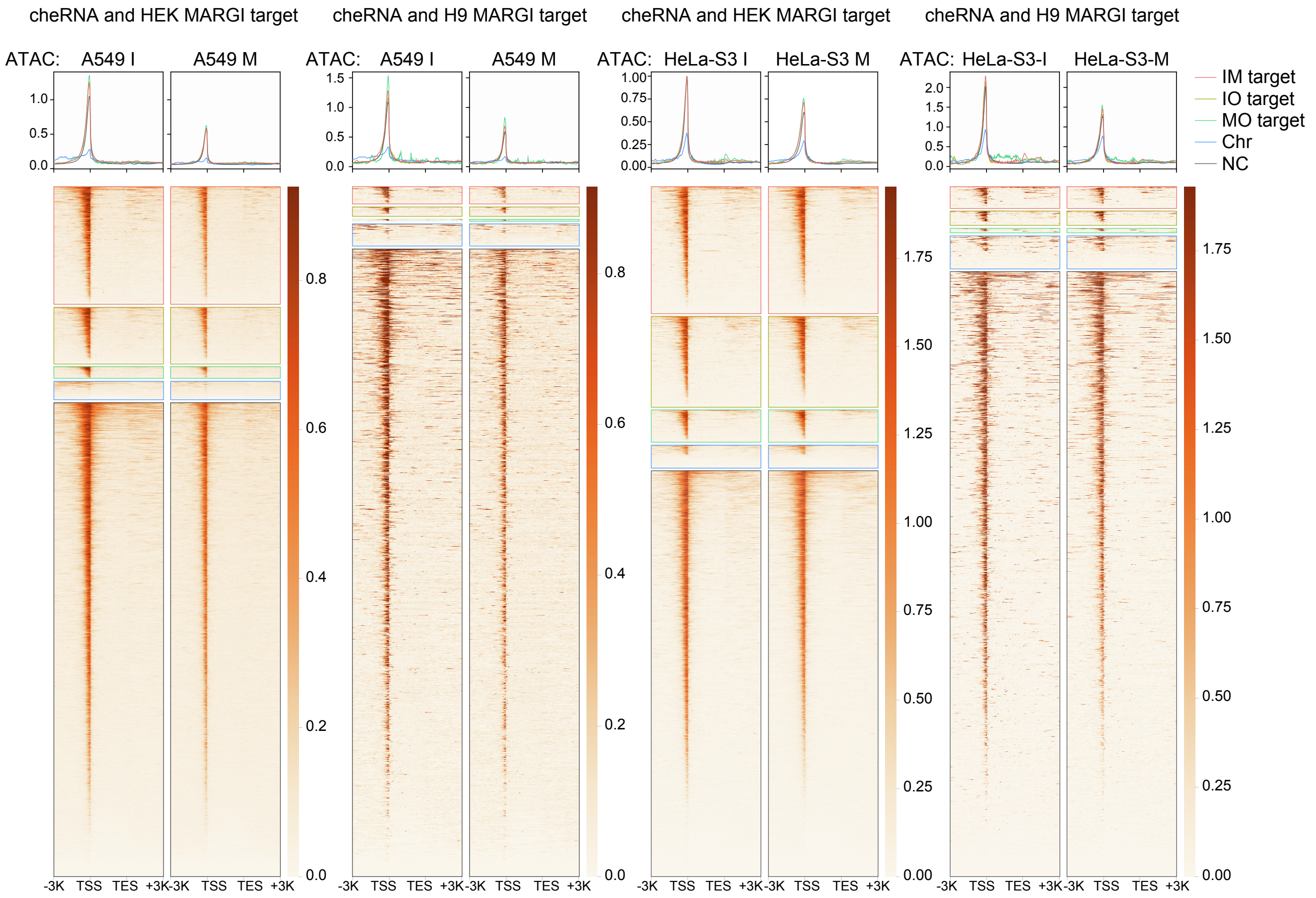

cheRNA and HEK MARGI target
cheRNA and H9 MARGI target
cheRNA and HEK MARGI target
cheRNA and H9 MARGI target
ATAC: A549 I
A549 M
ATAC: A549 I
A549 M
ATAC: HeLa-S3 I
HeLa-S3 M
ATAC: HeLa-S3-I
HeLa-S3-M
IM target
IO target
MO target
Chr
NC
-3K
TSS
TES
+3K

Figure S8

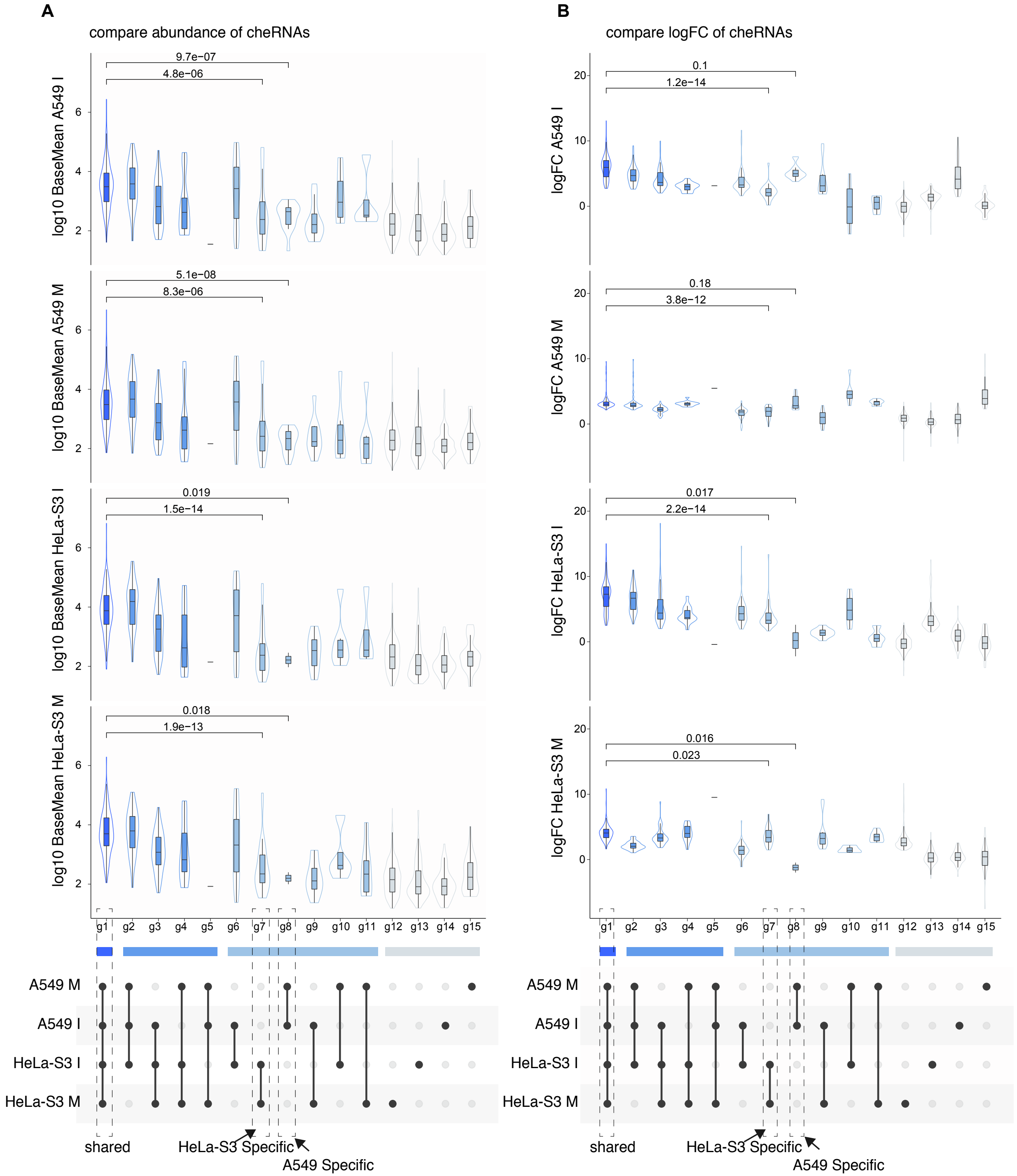
A
compare abundance of cheRNAs
B
compare logFC of cheRNAs
9.7e−07
4.8e−06
5.1e−08
8.3e−06
0.019
1.5e−14
0.018
1.9e−13
0.1
1.2e−14
0.18
3.8e−12
0.017
2.2e−14
0.016
0.023
log10 BaseMean A549 I
log10 BaseMean A549 M
log10 BaseMean HeLa-S3 I
log10 BaseMean HeLa-S3 M
logFC A549 I
logFC A549 M
logFC HeLa-S3 I
logFC HeLa-S3 M
g1 g2 g3 g4 g5 g6 g7 g8 g9 g10 g11 g12 g13 g14 g15
A549 M
A549 I
HeLa-S3 I
HeLa-S3 M
shared
HeLa-S3 Specific
A549 Specific
